# Study on droplet deposition characteristics and application of small and medium crown garden plants sprayed by UAV sprayer

**DOI:** 10.3389/fpls.2024.1343793

**Published:** 2024-05-17

**Authors:** Jinye Gao, Ping Bo, Yubin Lan, Liangchen Sun, Haiteng Liu, Xinlong Li, Guobin Wang, Huizheng Wang

**Affiliations:** College of Agricultural Engineering and Food Science, Shandong University of Technology, Zibo, China

**Keywords:** UAV sprayer, agriculture sector, application technology, garden plants, droplet deposition

## Abstract

The Unmanned Aerial Vehicle (UAV) sprayer has the advantages of high work efficiency, simple operation, and high safety factor, and has broad application prospects UAV sprayer are widely used in the agricultural field, and the application of UAV sprayer spraying technology in agriculture has provided convenience and increased profits for farmers, and has also become a research hotspot in the field of agriculture. In recent years, although research has been conducted on the feasibility and application effects of UAV sprayer spraying crown shaped plants, there have been no experiments or studies in the field of garden plants. This experiment conducted a droplet deposition experiment of UAV sprayer spraying garden plants, exploring the droplet deposition effect of UAV sprayer in the field of garden plants, and conducting experiments on the influence of spray volume and nozzle type on droplet deposition. The experimental results showed that the canopy performance of small and medium-sized garden plants was better at a flight altitude of 1.5m, a spray volume of 180L/hm^2^, and a flight speed of 2m/s. Reducing flight altitude, increasing spray volume, and reducing flight speed can improve the distribution of droplets in the canopy. This experiment lays the foundation for the application of UAV sprayer for the prevention and control of pests and diseases in garden plants, as well as for the application of growth regulators, and provides a basis for further innovative research in the field of garden plant application technology.

## 1 Introduction

Garden plants are an important component of urban construction and greening, which can effectively reduce urban noise pollution, improve air quality, and have a positive effect on improving the ecological environment (Peng et al., 2014; Lin, 2022). During the growth and development of garden plants, the occurrence of diseases and pests affects their growth and ornamental value. At present, the main way of spraying garden plants in China is to use ground spray. Some garden plants have complex terrain areas and tall trees, so it is difficult to use traditional spraying methods. The application of pesticide by the ground spray resulted in over spraying of pesticide, which caused a large amount of pesticide to be lost to the soil and air, causing environmental pollution (Li et al., 2020; Penney et al., 2021). According to reports, the use of pesticides in China is about 2.5 times the world level, and the area contaminated by pesticides is 1/10 of the arable area (Data from the Ministry of Ecology and Environment of the People’s Republic of China) (Chen SD. et al., 2020).

At present, the application of UAV sprayer in agriculture in China is in the fields of aviation pesticide application, crop management, and crop protection materials. The aerial pesticide application technology of UAV for plant protection has the advantages of water-saving and drug saving, high operation safety, high operation efficiency, and not limited by the operation terrain. It is particularly suitable for spraying garden plants such as small and medium-sized crowns, and spray operations in terrain where it is difficult for ground agricultural machinery to enter the ground (Lou et al., 2018; Yang et al., 2018; Zhiwei et al., 2019; Zheng et al., 2022; Chen et al., 2023).Expanding the application of UAV sprayer aerial spraying technology in the field of garden plants. Aviation spraying can reduce pesticide application by 15-20% through low spraying rate, which is an important technical support for China’s pesticide reduction planning (Chen SD. et al., 2020; Martinez-Guanter et al., 2020).

In recent years, research and application of UAV sprayer pesticide application technology have mostly focused on wheat, rice, and economic crops such as cotton (Yao et al., 2018; Kharim et al., 2019; Wang et al., 2019a; Yan et al., 2020; Huang et al., 2023). The application of UAV sprayer in agriculture has provided convenience and increased profits for farmers, and has also become a research hotspot in the field of agriculture. Wang et al. (2019b) used UAV sprayer to study the deposition of droplets on wheat crops, and the results showed that the deposition of droplets in the upper layer of wheat crop canopy was greater than that in the middle and lower layers. The spray system is the most important factor affecting the droplet deposition. Abd et al. (Kharim et al., 2019) used UAV to study the droplet deposition of liquid fertilizer on rice crops. The results showed that compared to higher flight speeds (4 and 6 m/s), the droplet uniformity and droplet deposition density were higher at lower flight speeds (2 m/s). Chen et al. (Chen SD. et al., 2020) conducted aerial spraying experiments on rice canopy with the same spraying rate and different droplet sizes using a multi rotor UAV sprayer and four TEEJET nozzles with different orifice sizes. The results indicate that droplet size is one of the important factors affecting droplet deposition and drift during pesticide spraying by UAV sprayer. When using UAV sprayer for ultra-low volume spraying, droplets with VMD less than 160 µm should be avoided. Lan et al. (2021) used a single rotor drone to study the influence of wind fields in the X, Y, and Z directions (X direction: parallel to the flight direction; Y direction: perpendicular to the flight direction; Z direction: perpendicular to the ground direction) on the deposition of liquid droplets in the rice canopy. The results showed that there were significant differences in the distribution of washing wind fields under different flight heights of UAV. The tested DJI T16 UAV sprayer has an optimal flight altitude of 2.0m, and the downwash field has a good improvement effect on droplet deposition. In summary, research on UAV sprayer in field crops has become relatively mature. Choosing appropriate operating parameters such as flight speed and altitude is an important factor in effectively depositing droplets on crops and improving the efficiency of UAV sprayer (Lan and Chen, 2018).

The application of UAV sprayer in fruit trees is mainly focused on economic crops such as fruit trees. The research mainly focuses on small crown apricots, citrus trees, and pear trees (Martinez-Guanter et al., 2020; Li et al., 2020; Guo et al., 2022), and there are few reports on the application of garden plants. Wang et al. (2022) studied UAV sprayer in hilly apple orchards and plain orchards respectively. The results showed that the application rate (APV) had a significant impact on the spray coverage parameters of the two orchards, while the impact of flight mode, intra row, inter row and vertical row was relatively limited, which were 600 and 857 L/ha, respectively. Li et al. (2020) used UAV sprayer to apply pesticides to apricot pests. The experiment mainly observed the uniformity and penetration of droplet deposition and verified the application of UAV sprayer on canopy plants. Zhang et al. (Pan et al., 2017). studied the effect of different canopy structures on droplet deposition in citrus trees. The results show that the droplet distribution performance of hedgerow and open canopy is better than that of round head canopy when UAV spray. Chen et al. (2017) explored the droplet deposition distribution effect and application prospect of small UAV sprayer on fruit tree spraying operation, and studied the influence of small UAV sprayer parameters on the droplet deposition distribution of orange tree canopy. The results indicate that the optimal operating parameters are nozzle flow rate of 1.0 L/min, operating height of 2.5 m, and operating speed of 4 m/s. The research mainly focuses on the feasibility and applicability of UAV sprayer on fruit trees, and the deposition of droplets on the canopy should be considered when spraying fruit trees. The tree shape of crops also affects the distribution of droplet deposition. The application of UAV sprayer on canopy plants requires selecting appropriate flight modes and parameters for different crops.

The flying altitude of the UAV sprayer spraying low and low field crops is 2m (Lou et al., 2018; Chen et al., 2021; Cavalaris et al., 2022), while the flying height of UAV sprayer on fruit trees needs to be reduced. Reducing the flying height can improve the distribution of droplets in the canopy (Wang J. et al., 2019; Zhu et al., 2019). Zhang et al. (Pan et al., 2016) studied citrus, considering the droplet deposition characteristics and spray uniformity, and the UAV sprayer performed better when operating on an open center shaped plant at a working height of 1.0 m. Wang et al. (Yi et al., 2002) found that adding plant adjuvant was positive in improving the distribution of droplet deposition in the canopy of fruit trees. Guo et al. (2022) also compared whether adding additives could increase the distribution of droplet deposition in southern pear trees, and the results were similar. Although research has been conducted on the feasibility and application effects of UAV sprayer spraying crown shaped plants, there have been no experiments or studies in the field of garden plants. This experiment conducted a droplet deposition experiment of UAV sprayer spraying garden plants, exploring the droplet deposition effect of UAV sprayer in the field of garden plants. The experiment conducted the influence of spray volume and nozzle type on droplet deposition. The experimental flight height was set at 1.5m and spray adjuvant were added to increase droplet deposition in the canopy, providing data support for the next step of UAV sprayer application technology in controlling garden plant diseases and pests.

## 2 Materials and methods

### 2.1 Experimental site

This experiment was conducted in a garden nursery in Zichuan, Zibo City, Shandong Province (117° 966723E, 36° 643452N), China, during was conducted in May 2023. Medium and small sized plants were selected as the experimental subjects according to the crown type of the experimental garden plants. The information on small and medium-sized garden plants is shown in **Table 1**.

**Table 1:** Information on small and medium-sized coronavirus test subjects.

	Latin name	Row spacing /m	Average plant height/m	Space/m	Test area/m^2^
Crown tree	*Malus’American’*	2	3.6	1.5	650
Small crown tree	*Euonymusjaponicus ‘Beihaidao’*	1.5	1.3	1	272

The meteorological conditions were recorded using a weather instrument (NK-5500, Nielsen Kellerman Co., Boothwyn, PA, 209 USA), with temperatures ranging from 25.9° C to 28.6° C, relative humidity ranging from 15.0% to 21.7%, and wind speeds ranging from 0.4 to 1.1 m/s.

### 2.2 Sprayers

This study used the DJI T30, a six-rotor UAV sprayer (Shenzhen DJI Innovation Technology Co., Ltd.), which can operate for approximately 20 minutes on a single charge. T30 can be used for fruit tree flight prevention, equipped with branch targeting technology, and can adjust the spraying elevation angle of the front and rear arms to allow droplets to penetrate the canopy along the oblique angle of the branch, ensuring uniform adhesion of the medicine. By using a handheld remote control to set flight speed, altitude, and spray width, route planning can be carried out in complex terrain areas using cloud platforms. It is also equipped with a centrifugal nozzle, high-precision flow meter, liquid level gauge, and dual view FPV camera. The main performance index parameters are shown in **Table 2**.

**Table 2:** Main Parameters of DJI T30 UAV sprayer.

Main parameter	Norms and numerical
Size/mm	2858×2685×790
Maximum load/L	30
Nozzle type	Centrifugal nozzle
Nozzles number	16
Spray amplitude/m	4-9m(12 nozzles)
Flight speed/m/s	7
Flight altitude/m	4500

### 2.3 Experimental design

**Figure 1:**
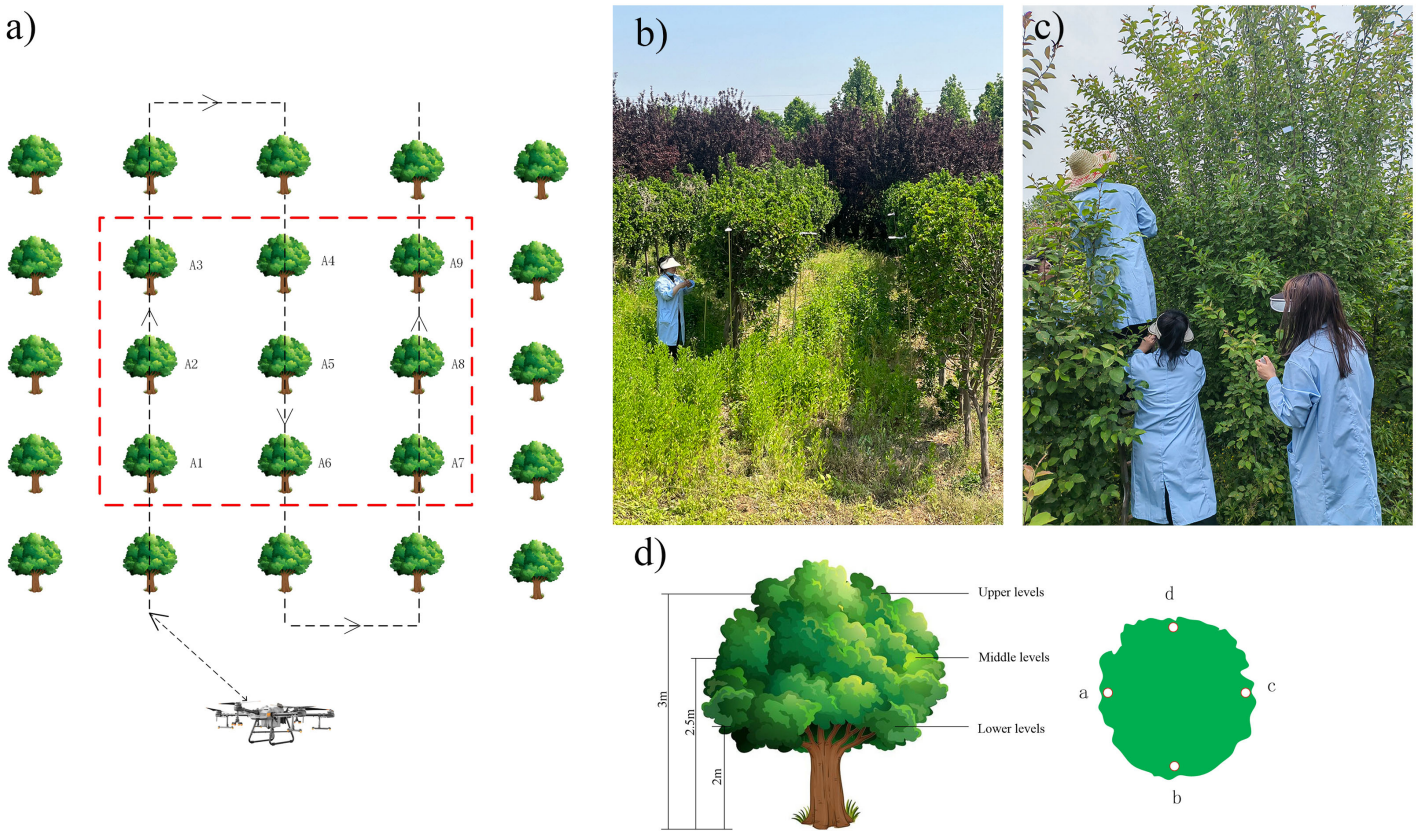
**(A)** Selection of target numbers and UAV sprayer operation routes; **(B)** Layout of sampling points; **(C)** Small Garden plant Hokkaido boxwood; **(D)** Medium-garden plant North American begonia.

Small Crown: Test area (the area is 17 m × 16 m) is divided into 9 treatments (**Table 3**), with 9 plants selected for each treatment (3 × 3) The target tree, as shown in **Figures 1A, B**, is sampled by selecting the middle layer of the target tree, and each tree is arranged with Kromekote^®^ cards (60mm × 20mm) in the east, south, west, and north directions.

**Table 3:** Experimental design of small and medium crown garden plants.

Small Crown			Medium Crown		
No. of test	Nozzle type	Spray volume/(L/hm^2^)	No. of test	Nozzle type	Spray volume/(L/hm^2^)
Treatment 1	SX11001VS	90	Treatment 1	SX11001VS	90
Treatment 2	SX11001VS	135	Treatment 2	SX11001VS	135
Treatment 3	SX11001VS	180	Treatment 3	SX11001VS	180
Treatment 4	XR110015VS	90	Treatment 4	XR110015VS	90
Treatment 5	XR110015VS	135	Treatment 5	XR110015VS	135
Treatment 6	XR110015VS	180	Treatment 6	XR110015VS	180
Treatment 7	IDK120-4	90	–	–	–
Treatment 8	IDK120-4	135	–	–	–
Treatment 9	IDK120-4	180	–	–	–

Medium Crown: Test area (the area is 26 m × 25m) is divided into 6 treatments (**Table 3**), with 9 plants selected for each treatment (3 × 3) Target tree, select the upper, middle, and lower layers of each tree for sampling, as shown in **Figures 1C, D**. Each layer is arranged in four directions: east, south, west, and north.

During the experiment, the flight height of the UAV sprayer was set to be 1.5m away from small and medium-sized garden plants. The flight speed of the DJI T30 was determined based on the spray volume of 90 L/hm^2^, 135 L/hm^2^, and 180 L/hm^2^, with corresponding flight speeds of 3 m/s, 2 m/s, and 1.5 m/s, respectively.

### 2.4 Measurement of droplet deposition

In this experiment, a 5 ‰ aqueous solution with a concentration of 85% temptation red dye was used as a tracer for spray testing. Purchased from Beijing Oriental Care Trading L., td., Beijing, China, is a water-soluble colorant commonly used in these types of studies. Studies have shown that the exposure distribution of temptation red is similar to that of pesticides. According to the Joint Expert Committee on Food Additives of the Food and Agriculture Organization/World Health Organization and the European Food Science Commission, it has high recovery rates, high photostability, and low acute toxicity in different species of animals (Cao et al., 2017; Chen SD. et al., 2020). After each treatment is completed, wait for the tracer on the Kromekote^®^ card to dry, collect the Kromekote^®^ card and bring it back to the laboratory for scanning using the DSG-1610 Epson scanner at a grayscale of 600 dpi, Using Deposit Scan (US Department of Agriculture) image processing software for image processing analysis, the distribution of droplets under different spraying operation parameters was obtained to obtain droplet coverage, droplet deposition density, and deposition amount (Zhu et al., 2011). The obtained data was analyzed.

### 2.5 Data analysis

This experiment used Excel (Microsoft Office 2021, Microsoft Corporation, Redmond, Washington, USA) and SPSS 26.0 software (SPSS Inc, an IBM Company, Chicago, IL, USA) to conduct statistical analysis on the obtained data, including the mean, standard deviation, and coefficient of variation of duplicate canopy data on the same group of trees. Univariate analysis of variance (one-way ANOVA) was used to test the significance of the differences at a significance level of 95%. The coefficient of variation is used to characterize the uniformity and penetration of droplet deposition between different collection points in the experiment (Zhu et al., 2011). The CV (coefficient of variation) of droplet deposition density at different canopy collection points of Hokkaido boxwood and North American crabapple is used to measure the uniformity of droplet deposition in the experiment. The CV of droplet deposition amount at sampling tree collection points is used to measure droplet deposition penetration. The coefficient of variation (Salyani and Cromwell, 1992; Sal and Fox, 1999; Hu et al., 2009) is:


$$\bar{X}=\frac{\sum^{}X}{n}$$

$$S=\sqrt{\frac{\sum^{}{\left(X-\bar{X}\right)}^{2}}{n-1}}$$

$$CV=\frac{S}{\bar{X}}\times 100\%$$

In the formula: $\bar{X}$ is the average value of droplet deposition; *X* is the droplet deposition value at each sampling point; *n* is the number of sampling points for each group of experiments; *S* is the standard deviation of the sample collected for the experiment. The smaller the coefficient of variation value, the better the uniformity of droplet distribution and penetration.

## 3 Results

### 3.1 Droplet coverage

**Figure 2:**
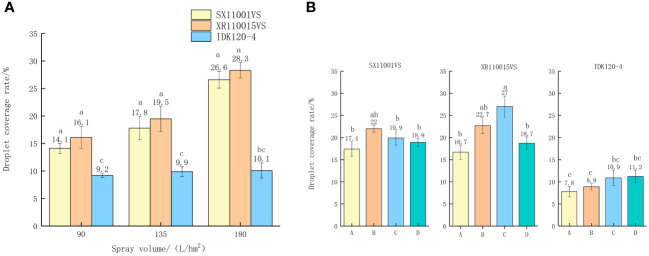
**(A)** Coverage rate of small Hokkaido boxwood; **(B)** Coverage rate of different types of nozzles at different points. The droplet density in the table is mean. Data followed by different small letters are significantly different among different treatments at p< 0.05 level by Duncan’s new multiple range test.

Droplet coverage is also an important parameter of droplet deposition and an important indicator of spray effect (Shan et al., 2021). **Figure 2** shows the droplet coverage of Hokkaido boxwood under different nozzle types and spray volumes. When the injection volume is 90, 135, and 180 L/hm^2^, the corresponding flight speeds are 3 m/s, 2 m/s, and 1.5 m/s, respectively. When using the same nozzle, the droplet coverage rate increases with the increase of spray volume. Among them, when the spray volume is 180 L/hm^2^ and the flight speed is 2.0 m/s, the droplet coverage rate is 26.6%, 28.3%, and 10.1%, respectively, with better performance; At the same time, under the same spraying volume, the nozzle XR1100015VS performs the best, with a coverage rate of 28.3%. The droplet coverage of nozzle IDK120-4 increases with the increase of spray volume, but the difference is not significant. **Figure 2B** shows the droplet coverage of four sampling points on the small Hokkaido boxwood canopy. The coverage rate of sampling point B of nozzle SX11001VS is significantly higher than other sampling points, at 22%, followed by sampling point C, at 19.9%. The droplet coverage of the nozzle SX11001VS is relatively average, and the difference in droplet coverage among the four sampling points is not significant. The droplet coverage rate at sampling point C of nozzle XR110015VS is the most significant, at 27%, which is significantly different from points A and D, followed by sampling point B, at 22.7%. The droplet coverage of nozzle IDK120-4 is relatively average, with sampling points C and D having droplet coverage rates of 10.9% and 11.2%, respectively. The extended range fan-shaped nozzle XR110015VS has better droplet coverage than the other two types of nozzles.

**Figure 3:**
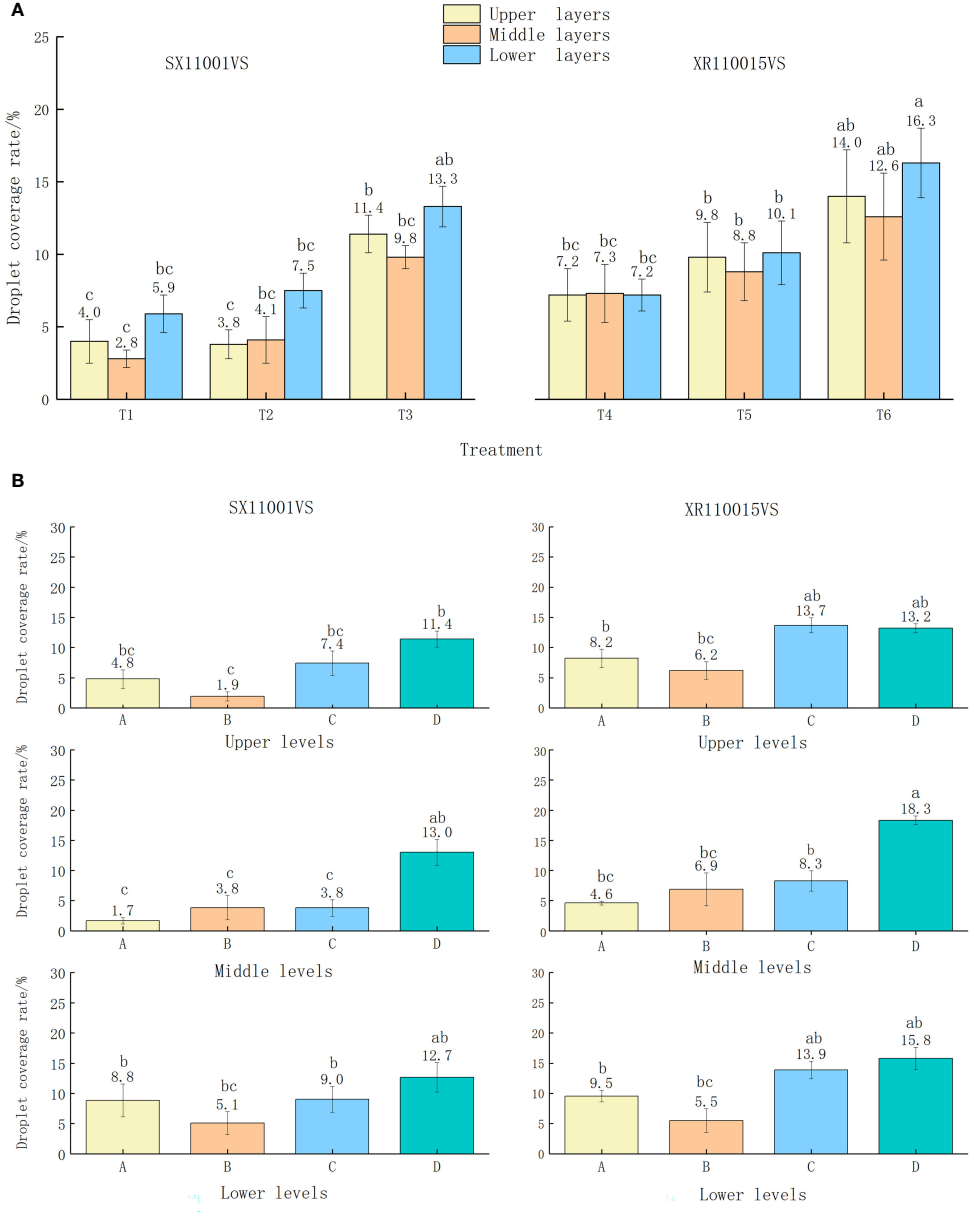
**(A)** Coverage rate of medium-sized North American crabapple in the upper, middle, and lower layers per treatment; **(B)** Coverage of different types of nozzles and sampling points in the upper, middle, and lower layers of medium-sized North American crabapple. The different lowercase letters in the figure indicate significant differences at the 0.05 level between different droplet sizes and spray volumes, as tested by Duncan's new complex range method.

It shows the droplet coverage of each treatment of North American begonia (**Figure 3**). The sampling points of North American begonia are divided into upper, middle, and lower layers according to the canopy. **Figure 3A** shows the droplet coverage of each treatment’s upper, middle, and lower layers. When the spray volume is 90, 135, and 180 L/hm^2^, the range of droplet coverage is 2.8-5.9, 3.8-7.5, 9.8-13.3, 7.2-7.3, 8.8-10.1, and 12.6-16.3%, respectively. When the spray volume is 90 L/hm^2^, the flight speed is 3.0 m/s, and the nozzle is XR110015VS, the droplet coverage rates in the upper, middle, and lower layers are relatively average, 7.2, 7.3 and 7.2%, respectively; When the spray volume is 180 L/hm^2^ and the flight speed is 1.5 m/s, the droplet deposition coverage is better. The droplet coverage range of SX11001VS is 9.8-13.3%, and the range of XR110015VS is 12.6-14%. The droplet coverage rate increases with the increase of the spray volume, and the droplet coverage result of the XR110015VS nozzle is consistent with that of the small-sized nozzle, which is superior to the sector pressure nozzle SX11001VS. **Figure 3B** shows the droplet coverage at the upper, middle, and lower points of nozzle SX11001VS and nozzle XR110015VS. The droplet coverage of nozzle SX11001VS is mainly concentrated at sampling point D in the upper, middle, and lower layers, with 11.4% in the upper layer, 13% in the middle layer, and 12.7% in the lower layer. The coverage of points A, B, and C in each layer varies greatly, with a coverage rate of 1.7% for sampling point A in the middle layer and 1.9% for sampling point B in the upper layer, The droplet coverage of nozzle XR110015VS is mainly concentrated in sampling points C and D in the upper and lower layers, and in sampling point D in the middle layer.

### 3.2 Droplet deposition density and uniformity

**Figure 4:**
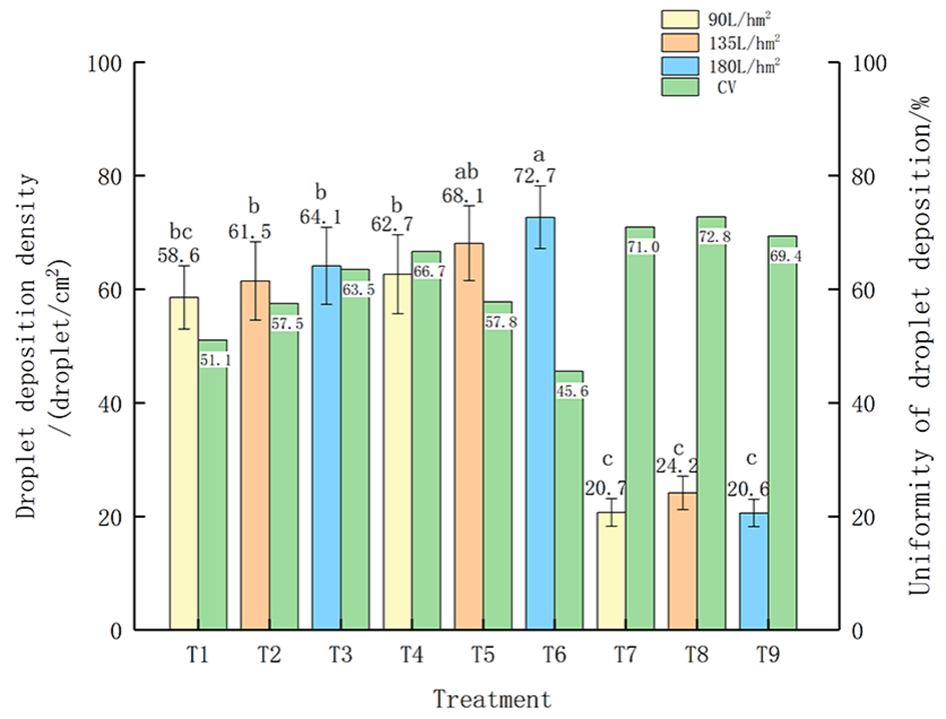
Droplet Deposition Density and Uniformity of Small Hokkaido Poplar. The different lowercase letters in the figure indicate significant differences at the 0.05 level between different droplet sizes and spray volumes, as tested by Duncan's new complex range method.

Uniformity refers to the uniformity of droplet deposition distribution at different levels along the height of crop growth, measured by the CV of droplet deposition density. Uneven droplet distribution can reduce the application quality of pesticides; therefore, droplet deposition density and uniformity are key factors for optimal pesticide use (Chen SD. et al., 2020). **Figure 4** shows the droplet deposition density and uniformity of small Hokkaido boxwood. T1, T2, and T3 are nozzle SX11001VS, T4, T5, and T6 are nozzle XR110015VS, and T7, T8, and T9 are nozzle IDK120-4. Under the same nozzle, the droplet deposition density also increases with the increase of spray volume, but the difference in nozzle SX11001VS is not significant. Among them, when the spray volume is 180 L/hm^2^ and the flight speed is 1.5 m/s, the droplet deposition density performs best for T3 and T6, 64.1 deposits/cm^2^ and 72.7 deposits/cm^2^, respectively. From the perspective of droplet deposition uniformity, when the spray volume of T6 is 180 L/hm^2^ and the flight speed is 1.5m/s, the coefficient of variation of droplet deposition uniformity is small, indicating that this treatment has good uniformity of droplet deposition.

**Figure 5:**
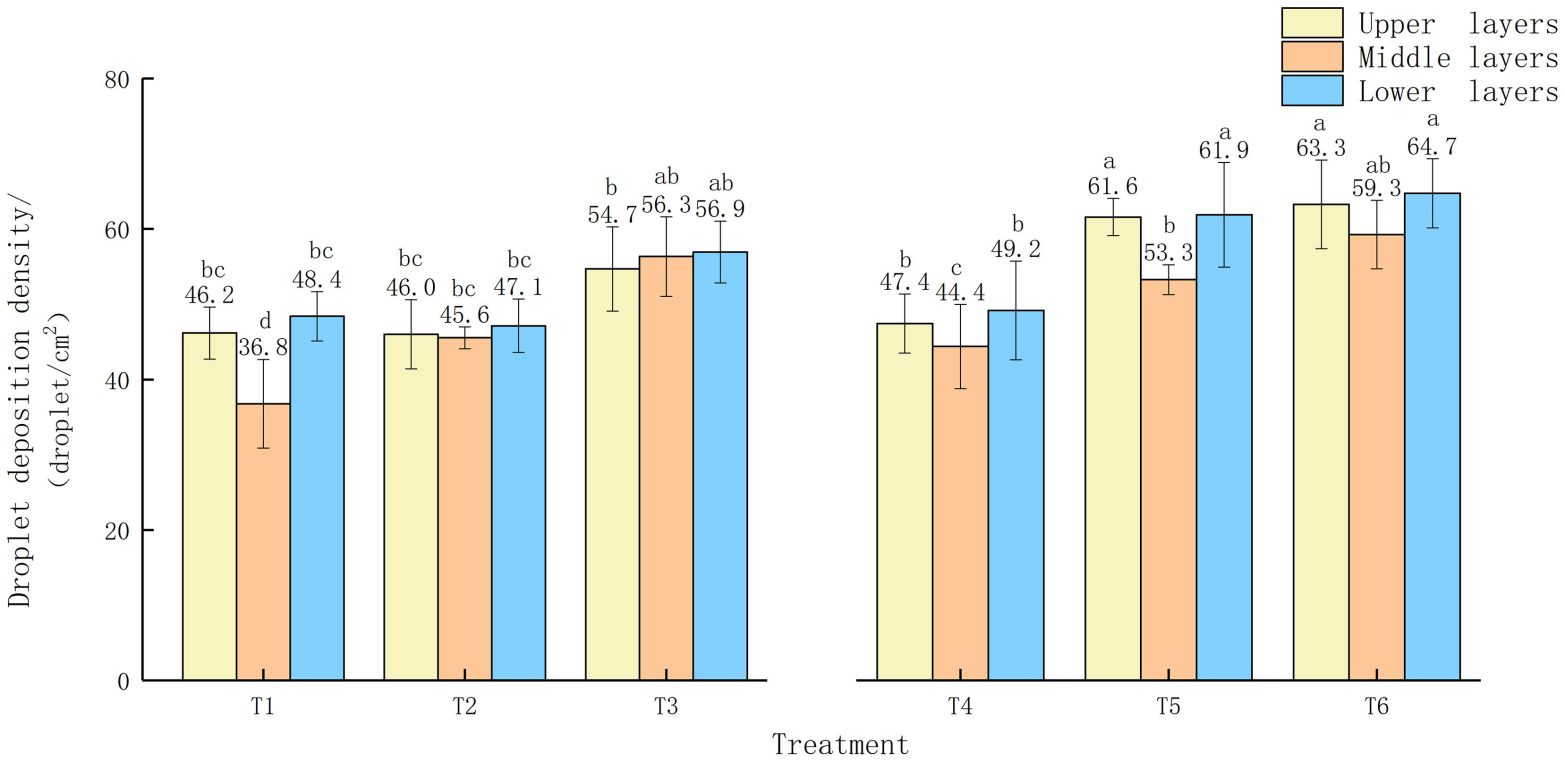
Droplet deposition density in the upper, middle, and lower layers of medium-sized North American crabapple under each treatment. The different lowercase letters in the figure indicate significant differences at the 0.05 level between different droplet sizes and spray volumes, as tested by Duncan's new complex range method.

**Figure 6:**
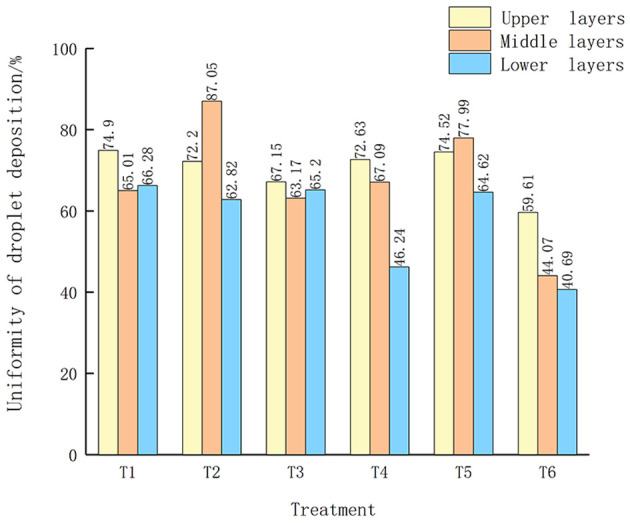
Uniformity of droplet deposition in medium-sized North American crabapple. The different lowercase letters in the figure indicate significant differences at the 0.05 level between different droplet sizes and spray volumes, as tested by Duncan's new complex range method.

The show the experimental results of droplet deposition density and uniformity of the medium-sized garden plant North American Begonia (**Figure 5**). The T1 and T4 shown in **Figure 5** show that when the spray volume is 180 L/hm^2^ and the flight speed is 1.5 m/s, the average droplet deposition densities on the upper, middle, and lower layers of North American crabapple are 54.7 and 56.3 deposits/cm^2^, 56.9 and 63.3 deposits/cm^2^, 59.3 and 67.4 deposits/cm^2^, respectively, which are basically better than other flight operation parameters with spray volume, The droplet deposition density of nozzle XR110015VS is better than that of nozzle SX1100VS. The distribution of droplets in the canopy of medium-sized North American crabapple shows a clear pattern, with a trend of droplet coverage, droplet deposition density, and droplet deposition amount in the lower layer of the tree canopy>upper layer>lower layer. Especially, the droplet coverage and droplet deposition density in the upper and lower layers of the tree canopy are significantly higher than those in the middle layer. UAV sprayer mainly deposit droplets on the upper and lower branches and leaves of open tree crowns. As shown in **Figure 6**, the CV of T6 is relatively small compared to other treatments, and the uniformity of the upper, middle, and lower layers is relatively stable. The results are similar to the uniformity of small droplet deposition density.

### 3.3 Droplet deposition and penetration

The deposition number of small droplets is shown in **Table 4**. It can be seen that as the spray volume increases, the surface deposition amount of Hokkaido boxwood gradually increases, with a unit area deposition amount of 0.68 and 1.25 respectively μL/cm^2^, 0.62 and 1.58 μL/cm^2^,0.27 and 0.37 μL/cm^2^. The average droplet deposition of nozzle XR110015VS is the best at a spray volume of 180 L/hm^2^, which is 3.23 μL/cm^2^, followed by the spray nozzle SX11001VS with a droplet deposition of 2.73 μL/cm^2^ at a spray volume of 180 L/hm^2^. The deposition amount of medium-sized droplets is shown in **Table 5**. The deposition amount of droplets in the lower canopy layer of nozzle SX11001VS at the spray volume of 90, 135, and 180 L/hm^2^ is 0.34, 0.42 and 1.3 respectively μL/cm^2^, The droplet deposition in the lower canopy layer of nozzle XR110015VS at a spray volume of 90, 135, and 180 L/hm^2^ is 0.91, 0.82, and 1.75 respectively μL/cm^2^, The droplet deposition rate of the extended range fan-shaped nozzle XR110015VS is higher than that of the fan-shaped pressure nozzle, and the droplet deposition rate of the two types of nozzles with a spray volume of 180 L/hm^2^ is the best. Research has shown that increasing the amount of spray and reducing flight speed can improve the distribution of droplets in the canopy, which is consistent with the results of this experiment (Chen et al., 2016; Wang J. et al., 2019; Chen et al., 2021).

**Table 4:** Small Hokkaido boxwood.

Nozzle type	Droplet deposition amount/(μL/cm^2^)		
	90 L/hm^2^	135 L/hm^2^	180 L/hm^2^
SX11001VS	0.80 ± 0.1	1.48 ± 0.23	2.73 ± 0.47
XR110015VS	1.03 ± 0.27	1.65 ± 0.22	3.23 ± 0.35
IDK120-4	0.62 ± 0.6	0.89 ± 0.84	1.26 ± 0.39

The deposition values are presented as means ± SD.

**Table 5:** Medium North American Begonia.

Nozzle type	Droplet deposition amount/(μL/cm^2^)								
	90 L/hm^2^			135 L/hm^2^			180 L/hm^2^		
	Upper levels	Middle levels	Lower levels	Upper levels	Middle levels	Lower levels	Upper levels	Middle levels	Lower levels
SX11001VS	0.23± 0.09	0.15± 0.04	0.34± 0.10	0.40± 0.24	0.26± 0.13	0.42± 0.08	0.95± 0.38	0.73± 0.25	1.30± 0.26
XR110015VS	0.43± 0.17	0.49± 0.19	0.91± 0.37	0.73± 0.37	0.52± 0.18	0.82± 0.21	2.25± 0.29	1.39± 0.42	1.75± 0.11

Based on the combination of droplet deposition density, droplet deposition amount, and droplet penetration (**Table 6**), it can be seen that when XR110015VS sprays a liquid volume of 180 L/hm2, operates at a height of 1.5m, and operates at a speed of 1.5 m/s, the penetration rates of small and medium-sized droplets are 69.42% and 67.41%, respectively. The droplet deposition density is high and the penetration rate is good, which can be considered as the better operation method for this experiment. This indicates that an increase in spray volume can effectively improve the penetration of droplets and achieve higher deposition. Zhang et al. (Pan et al., 2016) studied the influence of different canopy structures of citrus trees on droplet deposition. When spray by UAV sprayer, the droplet distribution performance of hedgerow and open canopy is better than that of round headed canopy, which is similar to the droplet deposition results of medium-sized begonia.

**Table 6:** Penetration of droplet deposition.

Nozzle type	Small Hokkaido boxwood/%			Medium North American Begonia/%		
	90L/hm^2^	135L/hm^2^	180L/hm^2^	90L/hm^2^	135L/hm^2^	180L/hm^2^
SX11001VS	78.82	81.08	88.06	76.15	77.14	74.84
XR110015VS	88.45	81.52	70.42	77.86	72.37	67.61
IDK120-4	99.09	91.03	90.23	–	–	–

## 4 Discussion

During the spraying operation of UAV sprayer, most of the spray chemicals are deposited on the top of the crop canopy in the field, which makes it impossible to control the diseases and pests in the lower layer, reducing the use rate of chemicals (Chen SD. et al., 2020). The amount of droplet deposition determines the effect of pest control, mainly including the droplet deposition density, uniformity and the amount of droplet deposition per unit area. The droplet deposition density and uniformity are important references and bases for improving the spray quality and pesticide utilization efficiency (Ru et al., 2012; Liao et al., 2015).

Results of the deposition coverage of small Hokkaido boxwood droplets at various locations, it can be seen that the sampling points with higher droplet coverage are B and C, mainly in the southwest direction. This may be due to the coincidence of the DJI T30 UAV sprayer with the northeast wind direction during operation, resulting in lower droplet coverage at sampling point A. Wang et al. (2016) explored the influence of flight mode, flight parameters, crosswind and other factors on the mass balance distribution of UAV spray droplets and the distribution of rotor downwind flow field. The greater the crosswind speed, the more concentrated the droplets are in the downwind direction. The droplet deposition effect of nozzle IDK120-4 is poor, and the droplets deposited on the paper card are thick, possibly due to the fact that IDK is an anti-drift nozzle. The nozzle with coarse droplets can effectively reduce droplet drift, but the distribution of droplet deposition in the canopy is poor (Chen P. et al., 2020; Wang et al., 2020; Zhang et al., 2024). Therefore, medium-sized North American crabapple no longer uses the nozzle IDK120-4 for experimentation.

When holding paper cards in the middle layer of medium-sized North American crabapple, they are arranged in the horizontal or vertical direction of the branches, which may be the reason for the significant differences in each point. Richardson et al. (Richardson and Newton, 2000) believe that the probability of droplet deposition affecting leaves should be related to the vertical projection plane area of the plant or gap, and a canopy with horizontal leaves is more likely to effectively capture large droplets than a canopy with vertical leaves. Wang et al. (2019b) studied the influence of three spray volumes (9.0 L/hm^2^, 16.8 L/hm^2^ and 28.1 L/hm^2^) on the droplet coverage using UAV sprayer, and found that the spray coverage increased with the increase of spray volume, which was consistent with the results of this test.

The results of droplet deposition uniformity for small and medium-sized garden plants are similar, with the best droplet deposition effect achieved when the spray volume is 180 L/hm^2^ and the flight speed is 1.5 m/s. The uniformity of droplet deposition in the upper and lower layers of medium-sized North American crabapple is relatively stable between each treatment, while the density and uniformity of droplet deposition in the middle layer exhibit instability; Based on the droplet deposition results of small garden plants, it can be seen that it is not related to the type of nozzle used. It may be due to the obstruction of upper leaves and branches in the middle layer, which makes droplet deposition more easily captured by the upper canopy of crops during UAV sprayer flight operations. As there is no obstruction above the lower canopy, droplets are also more likely to fall on the droplet collection paper cards arranged in the lower layer.

This study is based on the exploratory application of UAV sprayer in the deposition characteristics of droplets on small and medium-sized garden plants. This study aims to seek optimal flight operation parameters, increasing the penetration rate of droplets into the canopy of garden plants, and improving the deposition distribution of specific effective droplets at different levels of crop growth height, in order to improve the utilization rate of specifications When the spraying volume is 180 L/hm^2^ and the operating speed is 1.5 m/s, the droplet deposition effect of small and medium-sized garden plants is the best. During flight operations, it is necessary to analyze and adjust flight operation parameters according to the growth trend of garden plants. These data can provide data support for the application of UAV sprayer in garden plant spraying technology.

## 5 Conclusions

This study tested the effects of two types of nozzles and three spray volumes on droplet deposition characteristics in small and medium-sized garden plants. The results of the UAV sprayer spraying experiment on garden plants showed that the appropriate nozzle type improved the deposition of droplets on the canopy. In this experiment, the nozzle XR110015VS>SX11001VS>IDK120-4. The best droplet deposition effect is achieved when the spray volume is 180 L/hm^2^, the working height is 1.5m, and the working speed is 1.5m/s. The coefficient of variation for small droplet uniformity is 45.6%, while for medium droplet uniformity, the upper, middle, and lower layers are 59.6%, 44.1%, and 40.7%, respectively. The penetration rates for small and medium droplets are 70.42%, and 67.61%, respectively.

At present, there are few reports on the research on the effect of spray on garden plants by using UAV sprayer. This paper has conducted a preliminary exploration on the effect of spray in canopy space by using UAV sprayer on small and medium-sized garden plants, laying a foundation for the application of UAV sprayer to garden plants to prevent diseases and pests and to apply growth regulators, and providing a basis for further research on the application technology innovation in the field of garden plants.

## Data availability statement

The raw data supporting the conclusions of this article will be made available by the authors, without undue reservation.

## Author contributions

GJ: Conceptualization, Formal analysis, Investigation, Methodology, Software, Writing – original draft, Writing – review & editing. PB: Conceptualization, Data curation, Investigation, Writing – review & editing. YL: Project administration, Supervision, Writing – review & editing. LS: Writing – review & editing. HL: Investigation, Visualization, Writing – review & editing. XL: Data curation, Formal analysis, Writing – review & editing. GW: Investigation, Methodology, Writing – review & editing. HW: Conceptualization, Investigation, Methodology, Writing – review & editing.
